# Cellular and cytokine-dependent immunosuppressive mechanisms of grm1-transgenic murine melanoma

**DOI:** 10.1007/s00262-012-1290-9

**Published:** 2012-06-07

**Authors:** Miriam Alb, Christopher Sie, Christian Adam, Suzie Chen, Jürgen C. Becker, David Schrama

**Affiliations:** 1 Department of Dermatology, University Hospital of Würzburg, Würzburg, Germany; 2 Present Address: Klinikum Rechts der Isar, Department of Neurology, Technical University Munich, Munich, Germany; 3 Department of Chemical Biology, Susan Lehman Cullen Laboratory of Cancer Research in the Ernest Mario School of Pharmacy, Rutgers University, Piscataway, NJ USA; 4 Division of General Dermatology, Department of Dermatology, Medical University of Graz, Auenbruggerplatz 8, 8036 Graz, Austria

**Keywords:** Melanoma, Immune suppression, Tumor-draining lymph node, Regulatory T cell

## Abstract

Grm1-transgenic mice spontaneously develop cutaneous melanoma. This model allowed us to scrutinize the generic immune responses over the course of melanoma development. To this end, lymphocytes obtained from spleens, unrelated lymph nodes and tumor-draining lymph nodes of mice with no evidence of disease, and low or high tumor burden were analyzed ex vivo and in vitro. Thereby, we could demonstrate an increase in the number of activated CD4^+^ and CD8^+^ lymphocytes in the respective organs with increasing tumor burden. However, mainly CD4^+^ T cells, which could constitute both T helper as well as immunosuppressive regulatory T cells, but not CD8^+^ T cells, expressed activation markers upon in vitro stimulation when obtained from tumor-bearing mice. Interestingly, these cells from tumor-burdened animals were also functionally hampered in their proliferative response even when subjected to strong in vitro stimulation. Further analyses revealed that the increased frequency of regulatory T cells in tumor-bearing mice is an early event present in all lymphoid organs. Additionally, expression of the immunosuppressive cytokines TGF-β1 and IL-10 became more evident with increased tumor burden. Notably, TGF-β1 is strongly expressed in both the tumor and the tumor-draining lymph node, whereas IL-10 expression is more pronounced in the lymph node, suggesting a more complex regulation of IL-10. Thus, similar to the situation in melanoma patients, both cytokines as well as cellular immune escape mechanisms seem to contribute to the observed immunosuppressed state of tumor-bearing grm1-transgenic mice, suggesting that this model is suitable for preclinical testing of immunomodulatory therapeutics.

## Introduction

The innate and adaptive immune system is not just able to recognize and destroy foreign pathogens, but also cells that have undergone malignant transformation [1]. Malignant cells present different types of antigens, that is, germ line antigens, differentiation antigens, or antigens of mutated or aberrantly expressed proteins. Via cross-presentation, these antigens are processed by antigen-presenting cells (APC) to stimulate adaptive immune responses, allowing cytotoxic CD8^+^ T cells (CTL) to recognize these tumor antigens in the context of MHC molecules just like foreign antigens [2]. Indeed, tumor antigen-specific CTL have been described in peripheral blood and in tumors of cancer patients, for example, melanoma patients [3]. Furthermore, tumor-infiltrating lymphocytes (TIL) in solid tumors are considered a positive prognostic indicator of patient survival [4, 5]. But despite the presence of tumor antigen-specific CTL, most of the patients succumb to the disease [6].

The coexistence of a tumor-specific immune response and tumor progression in the same individual suggested the induction of immune suppression or tolerance at the tumor site. Indeed, in the meanwhile, several immune escape mechanisms have been described. For example, tumors often escape immune surveillance by losing MHC class I or costimulatory molecules expression or by rendering APC and CTL anergic or suppressive [7–9]. In addition, tumor cells can release immunosuppressive cytokines such as interleukin-10 (IL-10) or transforming growth factor-β1 (TGF-β1), which can not only inhibit anti-tumor immune responses but also increase proliferation of tumor cells in an auto- and paracrine manner [10, 11]. Indeed, elevated plasma levels of these cytokines were found in cancer patients [12–14].

IL-10 and TGF-β1 are also able to induce regulatory T cells, the so-called induced regulatory T cells (iT_regs_), from naïve CD4^+^CD25^−^ T cells and thereby suppress the cytolytic activity of CTL [15–17]. Furthermore, IL-10 is able to inhibit dendritic cell-mediated priming of CD8^+^ T cells abrogating the induction of an anti-tumor CTL response [18]. The important role of TGF-β1 can be deduced from the observation that inhibition of TGF-β1 signaling in T cells can induce anti-tumor immunity [19]. Moreover, in many tumor entities, the recruitment of thymus-derived “natural” CD4^+^CD25^+^FoxP3^+^ regulatory T cells (nT_regs_) contributes to the immunosuppressive status [20].

In the last couple of years, several spontaneous melanoma mouse models have been described. In order to test whether such models indeed reflect the human situation and could thus be suitable to study immunotherapies preclinically, we scrutinized spontaneous immune responses against melanoma in the grm1-transgenic mouse model LLA-TG-3. The transgenic mouse line TG-3 was originally generated to study obesity in mice [21]. The insertion of a 2-kb DNA fragment termed “clone B” in these transgenic mice, however, lead to the spontaneous development of cutaneous melanoma [22]. Gene mapping experiments revealed that the inserted transgene caused a 70-kb deletion in intron 3 of the metabotropic glutamate receptor 1 (grm1), which leads to an ectopic expression of this receptor. A link between grm1 overexpression and the development of hereditary melanoma was demonstrated by the targeted expression of murine grm1 cDNA under a melanocyte-specific promotor (dopachrome tautomerase; DCT) [23]. The original transgenic TG-3 line was crossed with Balb/c mice to establish a transgenic line with hereditary spontaneous amelanotic cutaneous melanoma (termed LLA-TG-3) [24]. For mating of the F2 generation, albino mice carrying the transgene were selected. Thereafter, LLA-TG-3 mice were bred by brother–sister mating, and thus have a Balb/c-dominated genetic background. Notably, these mice largely resemble melanoma development in humans, with first visible tumor lesions at the ears, eye lids, and perianal region occurring at the age of 10–12 weeks.

Thus, this model allowed us to study the generic immune response at different time points during tumor progression. Thereby, we could demonstrate that the proliferative capacity and expression of activation markers of T cells were greatly diminished with increasing tumor burden. This was associated with increased T_reg_ numbers and expression of IL-10 and TGF-β1 in tumor-draining lymph nodes and at the tumor site.

## Materials and methods

### Mice

LLA-TG-3 mice were bred by brother–sister mating under SPF conditions in the University Hospital, Department of Dermatology in Würzburg, Germany, according to the animal care guidelines. LLA-TG-3 mice were categorized into three groups according to their tumor burden (no tumor: no visible lesions, age range: 4–8 weeks; low tumor burden: small tumors (2–3 per mouse; size range: approx. 60–100 mm^3^), age range: 5–7 months; high tumor burden: large tumors (2–5 per mouse; size range: approx. 100–160 mm^3^), age range: 7–10 months). C57Bl/6j mice were purchased from Harlan Laboratories (Eystrup, Germany) and are later on referred to as wild-type (wt) mice (age range: 2–8 months). They were housed in our animal facility until they could be used as age-matched controls for tumor-bearing LLA-TG-3 mice.

### Antibodies and reagents

The following antibodies (Abs) were used for flow cytometry: PerCP-Cy5.5 anti-CD4 (RM4-5), PE anti-CD8 (53-6.7), FITC anti-CD69 (H1.2F3) (all from BD Biosciences, Heidelberg, Germany), FITC anti-CD4 (RM4-5), APC anti-CD25 (PC61.5), and PE anti-FoxP3 (FJK16s) (all from eBioscience, Frankfurt, Germany). Purified anti-CD4 (RM4-5) and anti-CD8 (53-6.7) Abs were used for immunohistochemistry (both from BD Biosciences). Recombinant human IL-2 was purchased from Chiron GmbH (Munich, Germany), phorbol 12-myristate 13-acetate (PMA) was from Calbiochem (Darmstadt, Germany), and concanavalin A (Con A) was obtained from Sigma-Aldrich (Schnelldorf, Germany).

### Immunohistochemistry

Frozen sections were fixed in acetone for 10 min followed by blocking of endogenous peroxidase with REAL™ Peroxidase-Blocking solution (DAKO Deutschland GmbH, Hamburg, Germany). After washing the slides twice in PBS, they were stained using predetermined optimal concentrations of primary Abs for 20 min. The slides were washed again in PBS and stained for 15 min with Histofine^®^ Simple Stain Mouse MAX PO Rat (Nichirei Biosciences Inc., Tokyo, Japan). After a final washing step, the slides were incubated for 15 min with Vector^®^ NovaRED™ Substrate Kit for Peroxidase (Vector Laboratories, Wertheim, Germany). The reaction was terminated by incubating the slides in H_2_O for 5 min. Finally, the slides were counterstained with Mayer’s hematoxylin (DAKO).

### Preparation of single-cell suspensions

Single-cell lymphocyte suspensions from spleens were generated by cutting the spleens into pieces and passing them through a 100-μm cell strainer. Cells were collected by centrifugation and resuspended in complete medium (CM; RPMI 1640 (PAN Biotech, Aidenbach, Germany) supplemented with 10 % (v/v) heat-inactivated FCS (Biochrom AG, Berlin, Germany), penicillin (100 U/ml; PAN Biotech), and streptomycin (100 μg/ml; PAN Biotech). The cell suspension was gently overlayed onto Lympholyte M (Cedarlane Laboratories, Ontario, Canada) and centrifuged for 20 min at 720×*g*. Lymphocytes were collected from the interphase, washed once in CM, and resuspended in CM. Peripheral lymph nodes (axillary, cervical, inguinal, lumbal, and poplietal) were pooled from each animal according to their drainage status. Single-cell lymphocyte suspensions from peripheral lymph nodes were generated by cutting them into pieces and passing them into a 12 × 75 mm round-bottom BD™ Falcon tube with cell strainer cap. The cells were washed once in CM and resuspended in CM.

### Flow cytometry analysis

For multicolor flow cytometric analysis, single-cell suspensions (2 × 10^5^ to 1 × 10^6^ cells) were treated with purified anti-mouse CD16/32 (93; eBioscience) to block FcR binding and stained at 4 °C using predetermined optimal concentrations of Abs for 30 min. The LIVE/DEAD^®^ Fixable Dead Cell Stain Kit, Near IR-fluorescent reactive dye (Invitrogen Life Technologies, Darmstadt, Germany), was used for viability staining. For detection of CD4^+^CD25^+^FoxP3^+^ T cells, the Regulatory T cell staining Kit (eBioscience) was used according to the manufacturer’s instructions. Fluorochrome compensation settings were set up using the BD™ CompBeads Anti-Hamster/Rat Ig, κ/negative control (FBS) Compensation Particles Set (BD Biosciences) with single stainings of the antibodies described above. Cells with the forward and side light scatter properties of lymphocytes were analyzed using a FACSCanto flow cytometer (Becton–Dickinson GmbH, Heidelberg, Germany). Background staining was assessed using non-reactive, isotype-matched control Abs (BD Biosciences, eBioscience). Data analysis was performed using FlowJo (Tree Star, Ashland, USA).

### ^3^H-Thymidine incorporation assay (proliferation assay)

Freshly isolated lymphocytes (1 × 10^5^) were cultured for 96 h in U-bottomed 96-well plates in CM supplemented with IL-2 (500 IU/ml) alone or together with either PMA (200 nM) or Con A (5 μg/ml). Proliferation was determined by the incorporation of ^3^H-thymidine (0.5 μCi/well) for the last 18 h of culture. Cells were harvested, and the radioactivity was counted in a scintillation counter. All experiments were done in triplicate.

### RT-PCR and *real-time* PCR

RNA was prepared from approximately 5 × 10^6^ single-cell lymphocytes, 2 × 10^6^ cells of B10.BR melanocytes, and LLA-TG-3 tumor cell lines or 25 six-μm-thick frozen sections of LLA-TG-3 tumor samples using the peqGOLD Total RNA Kit (Peqlab, Erlangen, Germany). cDNA was synthesized using the SuperScript first-strand synthesis system for RT-PCR (Invitrogen). IL-10, TGF-β1, and hypoxanthine phosphoribosyltransferase (HPRT) mRNA levels were quantified by *real-time* PCR using the 7500 Fast Real-Time PCR System (Applied Biosystems, Darmstadt, Germany). Analyses were performed using primers (Sigma Aldrich), an internal fluorescent TaqMan^®^ probe specific to HPRT (Eurogentec, Cologne, Germany), the PCR Mastermix for Probe Assays low ROX (Eurogentec), and the ABsolute QPCR SYBR low ROX Mix (Thermo Fisher Scientific GmbH, Karlsruhe, Germany) for IL-10 and TGF-β1. Primer sequences of IL-10 and TGF-β1 are listed online on PrimerBank (Primer ID IL-10: 6754318a2; Primer ID TGF-β1: 6755775a2); HPRT primers and TaqMan^®^ probe were designed with Primer Express 3 (Applied Biosciences). The relative expression levels of IL-10 and TGF-β1, normalized to HPRT and relative to wt splenocytes (for cell lines relative to B10.BR melanocytes), were calculated as $2^{{ - \Updelta \Updelta C_{T} }}$ with ΔΔ*C*_*T*_ = (*C*_*T*_ target gene, sample − *C*_*T*_ HPRT, sample) − (*C*_*T*_ target gene, wt spleen − *C*_*T*_ HPRT, wt spleen). *C*_*T*_ is defined as the cycle when the threshold level of fluorescence is reached.

### Statistical analysis

All statistical analyses were performed using GraphPad Prism 5 software (GraphPad Software, La Jolla, USA). Column values of the no tumor, low and high tumor burden group were evaluated for normality depending on the group size using the D’Agostino and Pearson omnibus normality test or the Kolmogorov–Smirnov test. When columns passed the normality test, the unpaired two-tailed Student’s *t* test was performed for comparison of two groups, and for comparison of more than two groups, the one-way ANOVA test with Dunnett’s multiple comparison post hoc test comparing the no tumor with the low and high tumor burden group, respectively, was performed. In those cases where at least one column did not pass the normality test, non-parametric tests were performed; that is the Mann–Whitney *U* test for comparison of two groups, and for more than two groups, the Kruskal–Wallis test with Dunn’s multiple comparison post hoc test comparing the no tumor with the low and high tumor burden group, respectively. Differences were considered statistically significant when *p* < 0.05.

## Results

### Increase of activated T lymphocytes in tumor-burdened animals ex vivo

**Fig. 1:**
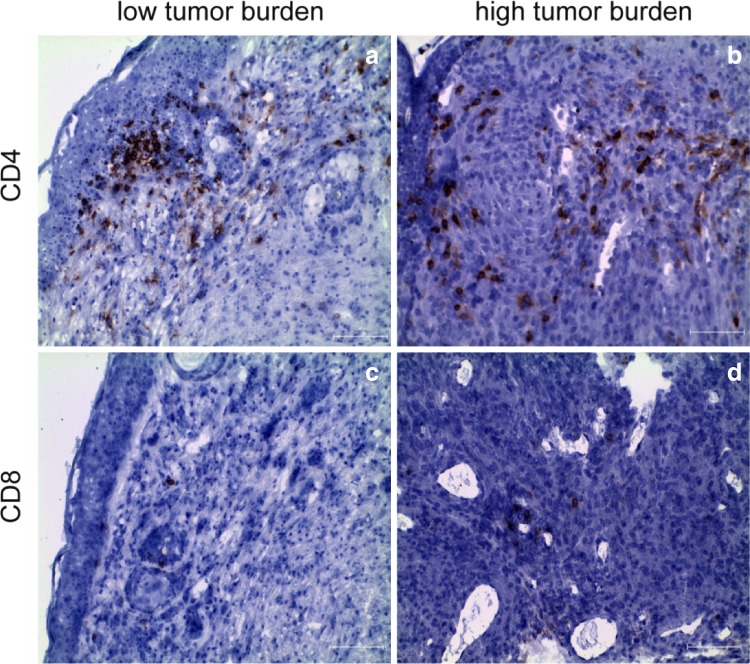
Tumor-infiltrating lymphocytes (TIL) in LLA-TG-3 tumors. **a**, **b** Showing CD4^+^ T cells in LLA-TG-3 tumors (red staining); **c**, **d** showing CD8^+^ T cells in LLA-TG-3 tumors (red staining). Nuclei were stained using hematoxylin. Scale bar: 100 μm. **a**, **c** Low tumor burden; **b**, **d** high tumor burden

As a first step to determine whether tumors in LLA-TG-3 mice evoke a cellular immune response, we characterized the T-cell infiltrate within the tumors by immunohistochemistry. These analyses demonstrated that lymphocytes infiltrating the tumor consisted mainly of CD4^+^ T cells with only few CD8^+^ T cells at the periphery of the tumor tissue (Fig. 1).

Next, we determined the ex vivo frequency of CD4^+^ and CD8^+^ T cells and their activation status in spleens and lymph nodes from wt and LLA-TG-3 mice. LLA-TG-3 mice were categorized into three groups according to their tumor burden, that is, no tumor, low or high tumor burden. Lymph nodes from tumor-bearing LLA-TG-3 mice were grouped into tumor-draining and non-draining according to their location to the tumor site (tdLN and ndLN, respectively).

**Fig. 2:**
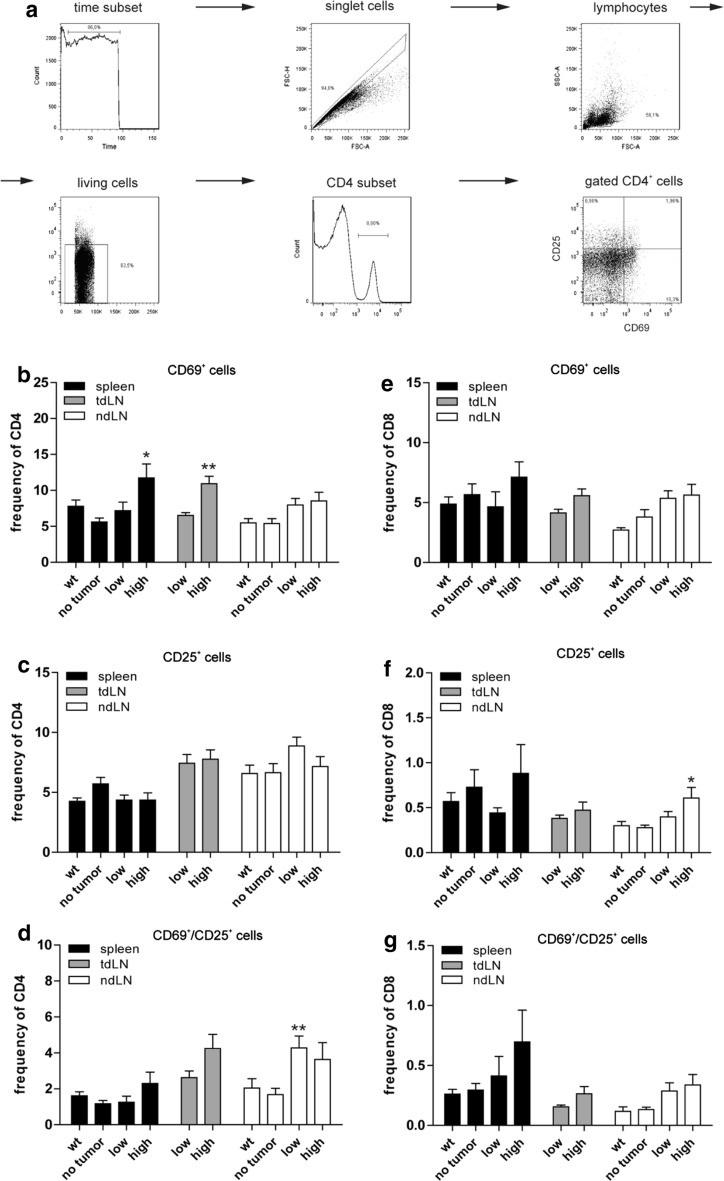
Gating strategy; CD4^+^ and CD8^+^ T-cell subsets ex vivo. **a** Gating strategy used for all flow cytometry data shown; **b**–**d** frequency of activated CD4^+^ T-cell subsets in spleen and lymph nodes of wild-type (wt) and LLA-TG-3 mice; **e**–**g** frequency of activated CD8^+^ T-cell subsets in spleen and lymph nodes of wt and LLA-TG-3 mice; *n* = 14 for wt mice; *n* = 10 for no tumor and low tumor burden LLA-TG-3 mice; *n* = 13 for high tumor burden LLA-TG-3 mice. Depicted are mean values + SEM. *p < 0.05; **p < 0.005

The gating strategy, which was used for all flow cytometry experiments, is shown in Fig. 2a. The frequencies of CD4^+^ and CD8^+^ lymphocytes in spleens and LN as determined by flow cytometry were similar among the groups (data not shown). However, the number of T lymphocytes expressing the activation markers CD69 (very early activation antigen, [25]) and CD25 (IL-2 receptor α chain, [26]) was in general higher in tumor-burdened animals compared to tumor-free LLA-TG-3 mice.

In this regard, statistical comparison between the no tumor, low and high tumor burden group revealed significant differences for CD69^+^ CD4^+^ lymphocytes in spleens and for both CD25^+^ CD8^+^ and CD69^+^/CD25^+^ CD4^+^ lymphocytes in ndLN (all *p* < 0.05; Kruskal–Wallis test). In detail, CD69^+^ CD4^+^ cells were more frequent in the high tumor burden compared to the no tumor group (*p* < 0.05; Fig. 2b); CD25^+^ CD8^+^ cells were more frequent in high tumor-burdened animals compared to tumor-free LLA-TG-3 mice (*p* < 0.05; Fig. 2f), and CD69^+^/CD25^+^ CD4^+^ cells were more frequent in the low tumor burden compared to the no tumor group (*p* < 0.005; all Dunn’s multiple comparison post hoc test; Fig. 2d). Similarly, the frequency of CD69^+^ CD4^+^ cells was also significantly elevated in tdLN of high tumor-burdened animals compared to the low tumor burden group (*p* < 0.005; unpaired two-tailed Student’s *t* test; Fig. 2b).

Taken together, we observed an increase of activated CD4^+^ and CD8^+^ lymphocytes in spleens and lymph nodes of tumor-bearing animals compared to tumor-free LLA-TG-3 mice.

### Diminished proliferative response upon mitogen stimulation with increasing tumor burden in LLA-TG-3 mice

**Fig. 3:**
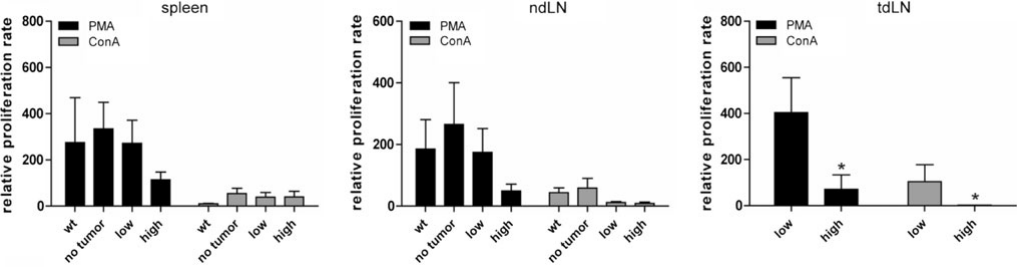
Proliferative response of lymphocytes upon mitogen stimulation. The proliferation rate of splenocytes and lymph node cells was analyzed using a ^3^H-thymidine incorporation assay after incubation for 4 days in medium supplemented with IL-2 (500 IU/ml) and PMA (200 nM) or ConA (5 μg/ml); *n* = 11 for wt mice; *n* = 9 for no tumor LLA-TG-3, and *n* = 10 for low and high tumor burden LLA-TG-3 mice. The proliferation rate was calculated relative to the proliferation rate in medium supplemented with IL-2 (500 IU/ml) only. Depicted are mean values + SEM. *p < 0.05

Since our T-cell analysis demonstrated an increase of activated T cells during tumor progression, we next tested the proliferative capacity of lymphocytes from LLA-TG-3 mice. To this end, lymphocytes were incubated in medium supplemented with IL-2 (500 IU/ml) alone (control) or with IL-2 and PMA (200 nM) or Con A (5 μg/ml) for four days. Proliferation was analyzed using a ^3^H-thymidine incorporation assay. The proliferation rate was calculated relative to the proliferation rate in medium with IL-2 only. In general, PMA was much more potent in stimulating proliferation than Con A. The proliferation rate in spleens and non-draining lymph nodes demonstrated already a trend toward reduced proliferation in the high tumor burden group (Fig. 3, *left and middle panel*). However, in tumor-draining lymph nodes of high tumor-burdened LLA-TG-3 mice, a significant decrease (*p* < 0.05; Mann–Whitney *U* test; Fig. 3, *right panel*) in the proliferation rate upon PMA and Con A stimulation was obvious compared to the low tumor burden group, suggesting especially in the tumor-draining lymph node an immunosuppressed state.

### Decreased expression of activation markers upon in vitro stimulation with increasing tumor burden in LLA-TG-3 mice

**Fig. 4:**
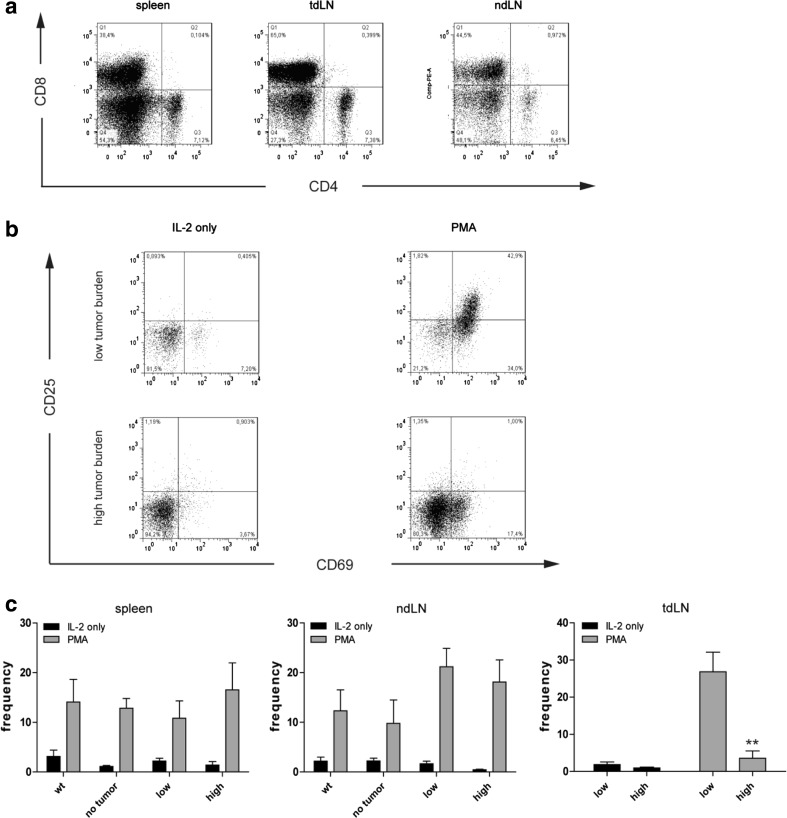
Frequency of CD4^+^ and CD8^+^ T cells upon IL-2 and PMA stimulation. **a** Representative dot plots of CD4 and CD8 stainings in spleen, tumor-draining (tdLN), and non-draining (ndLN) lymph nodes of a high tumor burden LLA-TG-3 mouse upon IL-2/PMA stimulation; **b** representative flow cytometry analysis of gated CD8^+^ T cells in tumor-draining LN of low and high tumor burden LLA-TG-3 mice upon stimulation with IL-2 (500 IU/ml) or IL-2 and PMA (200 nM). Numbers in top right quadrants indicate CD69^+^CD25^+^ cells; **c** the frequency of CD69^+^/CD25^+^ CD8^+^ T cells in spleen, tdLN, and ndLN of wt and LLA-TG-3 mice was analyzed as in **a**; *n* = 10 for wt mice; *n* = 6 for no tumor and low tumor burden LLA-TG-3 mice; *n* = 8 for high tumor burden LLA-TG-3 mice. Depicted are mean values + SEM. **p < 0.005

To analyze whether the reduced proliferation upon PMA simulation was due to impaired T-cell activation, we determined the activation status of T cells upon in vitro stimulation by flow cytometry. Lymphocytes incubated in IL-2-supplemented medium alone showed only minor differences in CD4^+^ and CD8^+^ T cell numbers among the groups (data not shown). Stimulation with PMA increased mainly the number of CD8^+^ T cells, but not of CD4^+^ T cells (Fig. 4a). However, the number of CD4^+^ lymphocytes expressing CD69 and CD25 was increased up to 10-fold upon PMA stimulation in spleens and lymph nodes among all groups (data not shown). In contrast, we observed a significant decrease of CD69^+^/CD25^+^ CD8^+^ T cells in tumor-draining lymph nodes (tdLN) of high tumor-burdened LLA-TG-3 mice compared to low tumor-burdened LLA-TG-3 mice in PMA-supplemented medium (*p* < 0.005; Mann–Whitney *U* test; Fig. 4b, c). However, in spleens and non-draining lymph nodes, the frequency of CD8^+^ T cells expressing both CD69 and CD25 increased equally among the groups upon PMA stimulation (Fig. 4c). Taken together, we observed that lymphocytes of wt and tumor-free LLA-TG-3 mice have a comparable upregulation of activation markers upon phorbol ester stimulation. High tumor-burdened LLA-TG-3 mice, however, are characterized by a reduced number of activated CD8^+^ T cells in tumor-draining lymph nodes upon in vitro stimulation. These findings confirm the immunosuppressed state of tumor-draining lymph nodes.

### Increased number of CD4^+^CD25^+^FoxP3^+^ T cells in lymph nodes of tumor-bearing LLA-TG-3 mice ex vivo

**Fig. 5:**
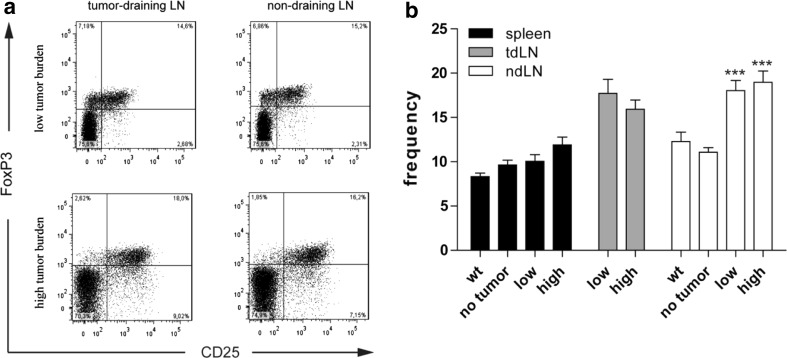
Frequency of regulatory T cells ex vivo (T_regs_; CD4^+^CD25^+^FoxP3^+^). **a** Representative flow cytometry analysis of gated CD4^+^ T cells in tdLN and ndLN of tumor-bearing LLA-TG-3 mice. Numbers in top right quadrants indicate CD25^+^FoxP3^+^ cells; **b** the frequency of T_regs_ was analyzed as in **a** in spleens and LN of wt and LLA-TG-3 mice ex vivo; *n* = 13 for wt mice; *n* = 10 for no tumor and low tumor burden LLA-TG-3 mice; *n* = 11 for high tumor burden LLA-TG-3 mice. Depicted are mean values + SEM. ***p < 0.0001

Regulatory T cells (CD4^+^CD25^+^FoxP3^+^; T_regs_) do not only inhibit autoreactive T cells to maintain immunological self-tolerance, but also anti-tumor immune responses in the tumor microenvironment [27]. As regulatory T cells (T_regs_) have been reported to be overrepresented in tumors and peripheral blood in cancer patients and to interfere with effector T-cell functions [20], we examined the frequency of T_regs_ in wt and LLA-TG-3 mice by flow cytometry. In spleens, we could not observe a significant change in T_regs_. In contrast, however, a significantly increased frequency of CD4^+^CD25^+^FoxP3^+^ T cells was present in lymph nodes of tumor-burdened animals compared to lymph nodes of tumor-free LLA-TG-3 mice (*p* < 0.0001 for both the low and high tumor burden compared to the no tumor group; one-way ANOVA with Dunnett’s multiple comparison post hoc test; Fig. 5a, b).

### Increased IL-10 and TGF-β1 mRNA levels in LLA-TG-3 mice with high tumor burden

**Fig. 6:**
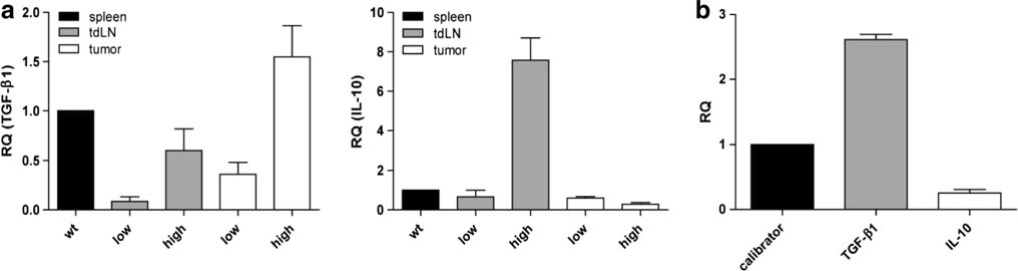
Relative mRNA expression of TGF-β1 and IL-10. **a** The mRNA levels of TGF-β1 (*left*) and IL-10 (*right*) relative to wt splenocytes were analyzed using qRT-PCR and cDNA from splenocytes, lymph node cells, and tumor samples of wt and LLA-TG-3 mice; *n* = 1 for wt mice; *n* = 3 for low tumor burden LLA-TG-3 mice; *n* = 4 for high tumor burden LLA-TG-3 mice; **b** the mRNA levels of TGF-β1 and IL-10 relative to the immortalized melanocyte cell line B10.BR (calibrator) were analyzed using qRT-PCR and cDNA from the two LLA-TG-3 tumor cell lines Nu2 and 4046T. Depicted are mean values + SEM

Since the increase in T_regs_ is a general event in tumor-bearing mice which cannot explain the decreased proliferation capacity of lymphocytes from mice with high tumor burden, we hypothesized that immunosuppression could be correlated with the expression of IL-10 and TGF-β1. Indeed, analysis of IL-10 and TGF-β1 expression status in lymph nodes and tumors using qRT-PCR revealed strong IL-10 and TGF-β1 mRNA expression in tumor-draining lymph nodes in high tumor-burdened LLA-TG-3 mice compared to the low tumor burden group (Fig. 6a). In the tumor tissue, however, we found only an increased TGF-β1 expression, whereas IL-10 expression was not elevated (Fig. 6a). This observation was in accordance with the mRNA expression levels of IL-10 and TGF-β1 in LLA-TG-3 tumor cell lines, that is, strong TGF-β1 expression and only moderate expression of IL-10 (Fig. 6b).

## Discussion

Most immune-modulating therapies are tested in preclinical mouse models. However, the most commonly used transplantation models do not reflect the normal tumor progression in humans, where tumors arise most often through a premalignant state over a longer period of time allowing for multiple immune escape mechanisms and immune editing [28]. Therefore, spontaneous tumor models may be more suitable to study immunotherapeutic approaches. Here, we analyzed spontaneously occurring immune responses during melanoma development in grm1-transgenic LLA-TG-3 mice. The spontaneous and stepwise development of amelanotic cutaneous melanoma in these mice largely resembles melanoma development in humans. We observed enhanced expression of the activation markers CD69 and CD25 on T lymphocytes ex vivo in tumor-bearing LLA-TG-3 mice compared to tumor-free LLA-TG-3 mice mainly in non-draining LN, but also in spleens and tumor-draining LN for CD69^+^ CD4^+^ T cells, indicating a systemic immune response activation caused by tumor development. Tumors themselves, however, were mainly infiltrated by CD4^+^ lymphocytes. From our data, we cannot conclude whether the immune response is directed against the tumor or triggered by tumor-induced inflammation or may even promote tumor growth [29]. Nevertheless, our findings are consistent with the situation reported for human cancers, that is, increase of activated lymphocytes in lymphoid organs and T-cell infiltrates in tumor tissue [30].

Interestingly, lymphocytes from tumor-burdened mice were functionally impaired in their proliferative response as measured by ^3^H-thymidine incorporation upon strong in vitro stimulation. The examination of activated T-cell subsets upon in vitro stimulation revealed a significant decrease in activated CD8^+^ T cells in tumor-draining LN of high tumor-burdened LLA-TG-3 mice. In contrast, the frequency of activated CD8^+^ T cells in the non-draining lymph nodes and the spleen upon in vitro stimulation was similar in LLA-TG-3 as well as in wt mice. Thus, the lymphocytes within these mice do not seem to be generally suppressed, arguing against a major impact of the age of these mice to the observed immune status. Indeed, when we selected the results from mice with similar age from the wt, low and high tumor burden group, the results were comparable to the results of the whole group (data not shown). Therefore, these findings suggest an active immunosuppression through the tumor itself or recruited regulatory mechanisms. Again, this is in accordance with the immunosuppressed state of lymphocytes in tumor-draining LN and peripheral blood of cancer patients [31, 32]. Recently, melanoma-induced suppression of T-cell proliferation, irrespective of antigen specificity, was also reported for B16F10 tumor-bearing mice [33].

Impaired effector T-cell functions are often associated with increased numbers of CD4^+^CD25^+^FoxP3^+^ regulatory T cells in tumor-draining LN and tumor tissue [34]. Natural thymus-derived CD4^+^CD25^+^FoxP3^+^ regulatory T cells (nT_regs_) have been intensely studied over the recent years. They play a crucial role in maintaining peripheral tolerance to self-antigens [35]. As many tumor antigens are self-antigens, for example, differentiation antigens like tyrosinase-related protein 2 (TRP-2), tumors can escape immune surveillance by recruiting nT_regs_ or by inducing regulatory T cells (iT_regs_) from naïve or effector T cells through release of IL-10 or TGF-β1 [15, 17]. Recently, it was also reported that absence of gamma-IFN-inducible lysosomal thiol reductase (GILT), which is involved in MHC class II restricted processing, leads to increase of TRP-1-specific T_regs_ and tolerance induction of TRP-1-specific T cells [36]. Indeed, the frequency of CD4^+^CD25^+^FoxP3^+^ regulatory T cells was increased in lymph nodes of tumor-bearing LLA-TG-3 mice compared to tumor-free LLA-TG-3 mice. This is consistent with the situation reported for melanoma patients, that is, elevated T_regs_ numbers in tumor-draining LN [37].

However, since increased T_reg_ numbers are likely not the sole immunosuppressive event during melanoma progression in these mice, we addressed IL-10 and TGF-β1 levels in tumor-draining LN and tumors of LLA-TG-3 mice, particularly, as both cytokines have been associated with both melanoma and T_reg_ development. Indeed, we detected increased IL-10 and TGF-β1 mRNA levels in tumor-draining LN of LLA-TG-3 mice. However, in tumors, only TGF-β1 expression was elevated. The same expression pattern, that is, strong TGF-β1 expression but only moderate IL-10 expression, was present in LLA-TG-3 tumor cell lines. This suggests a more complex regulation of IL-10 expression in this melanoma model. These findings confirm previous reports on TGF-β1 and/or IL-10-dependent immune escape mechanisms of melanoma [38]. Notably, both cytokines also influence lymphocyte development. Impaired anti-tumor immune responses have also been associated with increased levels of IL-10 and TGF-β1 in many cancer patients [18, 39, 40]. Moreover, TGF-β1 can directly inhibit expression of cytolytic genes in CTL [41]. However, not only tumor cells but also tumor-associated immunocompetent cells have been found to produce these immunosuppressive cytokines [42], a notion also corroborated by our results.

These findings offer several possible explanations for the observed immunosuppressed state in tumor-draining lymph nodes of LLA-TG-3 mice: (1) tumor-released TGF-β1 could actively suppress activation of CD8^+^ T cells in lymph nodes, (2) TGF-β1 and IL-10 in tumor-draining lymph nodes could induce regulatory T cells or other regulatory leukocyte subsets that suppress activation of CD8^+^ T cells either through cell–cell contact or release of additional immunosuppressive cytokines and/or (3) IL-10 could inhibit activation of CD8^+^ T cells through suppression of APC possibly by downregulation of B7 or MHC class II molecules [43]. We cannot, however, exclude the possible involvement of other immunosuppressive cell types such as Gr1^+^CD11b^+^ myeloid-derived suppressor cells (MDSC) [44] or CD8^+^CD122^+^ T cells (regulatory CD8^+^ T cells). Notably, the latter have been reported to suppress IFN-γ production and proliferation by CD8^+^CD122^−^ T cells in an IL-10-dependent manner [45].

In conclusion, we report here that both cellular as well as cytokine-dependent immunosuppressive mechanisms are employed during melanoma development in grm1-transgenic LLA-TG-3 mice. We observed many similarities to the immunosuppressive state present in melanoma patients, that is, functional impairment of T lymphocytes, increased numbers of T_regs_, and strong expression of IL-10 and TGF-β1 in tumor-draining lymph nodes. Therefore, the LLA-TG-3 melanoma model seems to be a suitable tool for testing immunotherapeutic approaches in a preclinical setting.

## Abbreviations

APC: Antigen-presenting cell
CM: Complete medium
Con A: Concanavalin A
CTL: Cytotoxic CD8^+^ T cell
DCT: Dopachrome tautomerase
FCS: Fetal calf serum
FoxP3: Forkhead/winged-helix transcription factor box P3
grm1: Metabotropic glutamate receptor 1
IL-10: Interleukin-10
i/nT_reg_: Induced/natural regulatory T cell
MDSC: Myeloid-derived suppressor cells
MHC: Major histocompatibility complex
ndLN: Non-draining lymph node
PMA: Phorbol 12-myristate 13-acetate
SEM: Standard error of the mean
tdLN: Tumor-draining lymph node
TGF-β1: Transforming growth factor beta 1
TIL: Tumor-infiltrating lymphocyte
TRP-2: Tyrosinase-related protein 2
wt: Wild-type
